# The Causes of Irregularity in the Development of the Teeth

**Published:** 1875-08

**Authors:** N. W. Kingsley

**Affiliations:** New York.


### AMERICAN JOURNAL

OF

### DENTAL SCIENCE.

Vol. IX. THIRD SERIES—AUGUST, 1875. No. 4.

### ARTICLE I.

### *The Causes of Irregularity in the Development of the Teeth.*

BY N. W. KINGSLEY, D. D. S., M D., NEW YORK.

The following excellent article from the Transactions of the N. Y. Odontological Society, is taken from the volume of Proceedings prepared by S. S. White, for *Cosmos.*

The subject of " The Treatment of Irregularities in the Development of the Teeth" is forcing itself upon the attention of the dentist with an alarming frequency. In no branch of practice does scientific knowledge seem to be sought with more avidity than in this direction. The tediousness of many of these cases; the inventive skill required to make successful appliances; the doubt always hanging over the final result; the lack of appreciation often of the highest order of professional skill; the absence of a prospective remunerative fee, as a stimulant; together with a want of knowledge of the causes operating to produce such an un-toward result, combine to make these cases the " *bete noir*" of dental surgery.

As the highest order of scientific investigation is now directed more to the prevention of disease than to its remedy, no more important subject can now engage our attention, than an inquiry into the origin of these deformities, and of some means to be used to prevent their recurrence in our offspring. And here, upon the very threshold of our research, we are confronted with the paucity of our knowledge, and the feebleness of our abilities; for science never has and never can discover the primary cause of any one of the phenomena of nature. With all her efforts, she has succeeded in penetrating only a little further into the unknown, leaving the origin still as dark and mysterious as ever. Nevertheless, there are operations in nature which, for the purpose of science, may be denominated primary causes, which may be discovered, measurably controlled, and their disastrous influences averted.

Many of the forms of irregularity are directly traceable to inheritance, and are transmitted peculiarities. Probably in a very large proportion of cases where the irregularity in a dental arch is confined to one or two teeth, the primary cause, so far as that individual is concerned, is an hereditary family peculiarity.

The teeth of every person possess more or less individuality and most of those peculiarities which stamp their individuality are inherited. The form and color of teeth, when not disturbed by abnormal influences, are derived from the same source.

Whenever we find any departure from what we are apt to regard as the typal form of each tooth, or any disproportion of size in their relations to each other, we shall be likely to find them peculiarities of descent.

The transmission by inheritance of a predisposition to a defect or a deformity, is the result of the same general law of nature which gives the form and features of progenitors to their offspring.

In the light in which we are now discussing this subject, it would be unprofitable to attempt to discover the cause of the minor peculiarities in dental development, which have clearly an origin in the ancestry of the individual. An investigation of this character would involve the discussion again of the "Origin of Species," and we should be compelled to admit,—as we have heretofore and must always hereafter,—that there lies a cause beyond all that we can comprehend, and which never can be reached by finite minds.

It is certainly a most wonderful subject for contemplation that, at some remote period in the history of our progenitors, nature departed from her normal type, and a dwarfed lateral incisor, a twisted canine, or an undeveloped bicuspid was the result; and following down the line of descent, we find precisely the same peculiarity appearing and reappearing; not always confined to the direct line, but continuing in the same family, by passing to the children of the brother or sister, and always presenting characteristics identical with the antecedent type.

"There is an ingrafted tendency in all living organized matter to reproduce itself."

It is yet to be determined whether the correction of the irregularities of the dental organs exercise any influence upon the offspring. That the correction of a congenital deformity in a parent will prevent its transmission, is no more to be supposed than would be the expectation that if we removed all the eyes from the present generation, the next would be born blind, excepting in so far that we have a right to expect that, in a transmitted deformity, nature will be making a constant effort to return to the normal type, while a created deformity would be by nature ignored.

Irregularities, either in the form of the arch or the position of the teeth, are very uncommon in the deciduous set. I have never seen an irregular arch in a child prior to the eruption of the permanent teeth, unless associated with and correlated to some other deformity. I have in a few instances seen a slight malposition of one or more of the incisors, sometimes of congenital origin, and sometimes the result of mischievous habits; as, for example, the two centrals may be pulled forward out of line by the prolonged use of an artificial nipple, sucking the thumb, or other similar habit. Of congenital deformities, it is rarely more than a trifling displacement of one or two of the incisors; but considering the temporary character of the deciduous teeth, and more especially the incisors, no irregularity in their position, that I have ever seen, can be regarded as having any material bearing upon the greater subject now under discussion.

They are to be classed as mere freaks of nature, not associated with nor indicating any other peculiarity in the child. Nor does it by any means prognosticate an irregularity in the development of the second set. This important fact cannot be too prominently borne in mind. The deciduous dental arch is always well formed, and the positions of the teeth are regular (mere freaks of nature excepted.) And from this perfectly symmetrical dental arch there develops with the growth of the permanent set some of the most astounding abnormalities.

These peculiarities of the permanent it is unnecessary to describe in detail. In the departure from symmetry they assume almost every variety of position, so that it would be almost impossible for the human mind to conceive of an irregular arrangement which would not find its counterpart in nature.

These variations are recognizable by every one of extended observation, and are deformities, because they are a greater or less departure from a normal standard. Such a standard cannot, in the very nature of things, be one shape to which all must conform or be classed as deformed.

Symmetry and harmony do not imply uniformity; and the dental arch may be developed up to the highest type of perfection, and yet there exists as great a variety of form as there would be in the faces of the aggregated beauties of the world.

Races, nations, and families are thus represented without deformity.

The normal type of the dental arch I conceive to be a regular line; the arch may be wider or narrower, varying somewhat in individuals or races, but the line would be an easy, graceful curve, without break or tendency to form an angle.

Within certain limits a narrow-dental arch, as associated with certain features, may become the very perfection of beauty, while with another form of head and face the wildest development may be equally pleasing. That which is recognized now as the standard or full measure of beauty, as well as of utility, is not unlike that which existed in the remotest historic ages, nor different from that which is now exhibited among all communities not degenerated by luxury or vice.

I have recently held a most interesting conversation with Dr. Nichols, who has spent twelve years in the Rocky Mountains and on the Pacific Coast, during which period he has examined the mouths of thousands of Indians and Chinese. He informs me that he never saw an instance of irregularity of the teeth in either of those races, with but one exception, and that a displaced canine in the mouth of a Chinese woman. The jaws of both races are universally well formed and amply developed. This is the evidence of an expert,—Dr. Nichols being a practitioner of dentistry. And this is also true of all semi-barbarous and savage races of good physical organization.

In 1864, Messrs. Cartwright and Coleman, of London, made an examination of some two hundred ancient skulls in the crypt of Hythe Church, Kent. These skulls, of which there is no authentic history further than that they have been there for centuries, were apparently of both sexes and all ages.

The maxillæ presented in all instances unusually well developed alveolar arches. The teeth were remarkable for regularity of position, only two deviations being noticed; one upper canine shut within the lower jaw on occlusion, and one bicuspid was turned upon its axis, and there might have been other slight irregularities which were unnoticed; but in no single instance was there anything seen approaching to that which, under the term "contracted arch," so commonly exists in the present day.

The average width of the dental arch in these skulls from the outside of the first molar to the corresponding point was two and a half inches.

In 1869, Mr. John B. Mummery, of London, contributed to the Odontological Society of Great Britain the most valuable paper on this subject which I have ever read. I accord more importance to his personal examinations than I do to the observations of any man not a practical dentist. The statements of all others, even those of ethnologists, being less precise, and more general in their character, must be accepted with some allowance. He examined all the available skulls of ancient races, and of modern uncivilized races, to the number of about three thousand, and tabulated more than one-half of them, which were classified as follows:

Ancient British, 203; Roman British, 143; Anglo-Saxon 76; and Ancient Egyptian, 26.

Of modern uncivilized races; North American, 145; Polynesian, 204; East Indian, 223; African, 438; and Australian, 165,

From a careful analysis of the measurements given in his tables, I find that the average width of the dental arch from first molar across to first molar, in the skulls of ancient races, was a trifle less than two and three-eighths inches; the same measurement of the uncivilized moderns showed an average width of a trifle above two and a half inches.

The narrowest measurement given by him of any skull of any race is two and one-eighth inches.

The highest average of any race is nearly two and three-quarter inches, and these belong to the New Zealander, the Fejee Islander, and the Ashantees.

The narrowest average was found among the Hottentots and Bushmen of South Africa.

In these tables we have abundant evidence that the full measure and type of both dental and maxillary arches has been sustained among all peoples of simple habits and thoughts in all ages.

The standard of normality of the dental arch is a curved line expanding as it approaches the ends, and the teeth all standing on that line.

Abnormality will include such a shape of the arch as is not in harmony with the surrounding features; all crowding and twisting; and all departures from a regular line in the positions of the teeth.

Almost the only answer received by the dental student as to the cause of these irregularities has been "premature extraction of the deciduous teeth," and consequent contraction of the jaw; and this answer has been almost universally accepted without a question as to its philosophy. A few facts have been correlated, and a conclusion as unscientific as it is erroneous arrived at; and to the anxious inquiry of the parent has come the charge made against some other dentist of gross malpractice.

It is only within a few years that any one has been bold enough to doubt the universally accepted theory which so glibly accounted for every presentation of abnormality. Among those who have pronounced this ground as untenable, and publicly given their arguments, may be mentioned Messrs. Cartwright and Tomes, of London, and Dr. Forbes, of St. Louis.

Their arguments are based upon the deductions of science and the observations of facts, and are also confirmed in the experience of others.

The premature extraction theory rests upon the supposition that the jaw-bone contrasts upon the removal of the deciduous teeth. The fact seems to have been entirely ignored that the teeth and alveolar processes are a superstructure of the jaw-bone; growing upon it; fulfilling their destiny, and passing away without disturbing the foundation much more than an oak disturbs the planet upon which it has been sustained.

There exists a period in the history of the maxillæ when it is itself an entity, and prior to hardly a trace of the subsequent superincumbent structures ; and in the ordinary course of nature there comes a period at the other end of life when equally all trace of dentition is gone and the maxillæ remain an undestroyed entity.

While the proofs are conclusive that the jaws are developed independently of the teeth and alveolar processes, and that no ordinary surgical interference with the teeth or processes impedes or impairs that development, and while it may also be true that all the primary teeth may be removed long anterior to the period of eruption of the permanent ones without retarding their development or impairing their regularity, the doctrine may still be correct that the too early extraction of some of the temporary teeth will be likely to result in a crowded and abnormal condition. This result is not as likely to show itself with any other teeth as with the canines.

Let us look carefully into the order of the eruption of the permanent set in the regular economy of nature. It will be admitted that for every individual tooth of the deciduous set there is a permanent one lying underneath it in process of formation, and that in due time a deciduous tooth will loosen and fall out, and a permanent one will take its place.

In a normal state there will occur no interference and no irregularity. The central incisors will first appear ; then the laterals ; after that the bicuspids ; and lastly the canines. Each predecessor of a permanent tooth will maintain its position until about the period of the emergence of its successor. There would be no contraction of the alveoli, because there would be no opportunity for it.

But taking now a disturbed, instead of a normal condition, let us see the result.

Remove for any cause one after another, or all at once, of the temporary set, and the period of development would not be interfered with, but the arrangement in the arch might be impaired.

The centrals would find a place without difficulty,—so would the laterals; the bicuspids would be pretty certain to have sufficient room, as their diameters are generally less than their predecessors; but when these teeth were all fully erupted, it would be found in a majority of cases at the present day that the first bicuspid and the lateral were nearly or quite in contact, filling the space destined for the permanent canine, which must now emerge either anteriorly or posteriorly to its true position, showing conclusively that if the alveolar arch had not contracted, the contiguous teeth had encroached upon the space destined for the canine, and forced it out of position. In either case it was the unquestioned result of the too early removal of the deciduous canines, for no one will doubt that, had the temporary canines been allowed to remain, they would have prevented contraction of the alveolus or an encroachment on their domain. It may be argued that if the original direction of the canine was correct, it would force itself between the lateral and bicuspid, thus making way for itself; but this is against the experience of nearly all observation, and very naturally so.

Admit the fact that from some cause or other the bicuspid and lateral come into contiguity, and from the tardiness of the erupting canine, it will find solid and unyielding roots to contend with, which will necessarily force it out of the dental arch.

Mr. Tomes relates a case in which he removed, for cause, from a child, all of her deciduous teeth prior to the eruption of any of their successors, so that for a time the gums were edentulous; nevertheless, in due time the permanent teeth developed in perfect regularity of sequence and of symmetry·

While Mr. Tome's report shows that no abnormality followed the removal of all the teeth at once, such a result is to be feared from such a practice, and most certainly will ensue if there be not a coincident, independent, and ample development of the maxillæ. Cases are coming constantly under our observation where from some cause the temporary canines have been sacrificed, the space closed up, and the permanent teeth malposed, with little hope of their ever assuming, unaided, their rightful positions.

From this there can be but one deduction,—which is, that whatever may be the inducement to remove any or all of the deciduous teeth prior to their period of shedding, the canines should be retained until there is ample evidence of their permanent successors.

How much of the malposition of the permanent teeth is due to the prolonged presence of the temporaries, is, I think, still an unsettled point.

Although absorption may be proved to be an independent process, and in no wise connected always with the progress of an erupting tooth, nevertheless, the cases are so common in which the absorbed portion of the deciduous tooth corresponds so accurately with the new crown, as to force upon us the conviction that it is influenced by it, and therefore that if the permanent tooth had persisted in the right direction the deciduous tooth would have become displaced.

The question very naturally arises, is the presence of the deciduous teeth the *cause* or the *effect* of the irregularity? If their presence be the cause of irregularities, then it is manifest that in this generation of malposed teeth it is our duty to anticipate the trouble, and at an early day remove them, before even it is possible for them to give a wrong direction to their successors. Whether their presence be the cause or the result of the malposition, this we do know, that when a permanent tooth has erupted, and its deciduous predecessor has not been removed, the immediate extraction of such tooth will go very far toward the complete correction of the deformity.

I do not see in this very common fact any greater evidence that the presence of the temporary tooth caused the irregularity than that the position and tendency of the permanent tooth was originally wrong. It is quite as reason able to adopt the latter view as to suppose that the function of absorption was arrested, and by that the tooth was turned aside.

In seeking information of the causes of irregularity more with a view of discovering some means of prevention than of correction, it was with some disappointment that I read Mr. Tome's explanation of certain abnormalities which he had observed, described and treated.

For example, in describing cases which are not uncommon where the upper front teeth project unduly, shutting over the lower lip, he says :—

"The deformity may result from excessive development of the alveolar processes of the anterior part of the upper jaw, but more commonly we shall find that the molar teeth are unusually short, thereby allowing the incisor teeth of the lower to press unduly upon the inclined lingual surfaces of the teeth of the upper jaw. The upper teeth yielding the pressure, are forced outward, and are retained in the malposition by the teeth which have led to the displacement. If, in cases resulting from the latter cause, the inquiry be extended to the condition of the lower jaw, it will be found that with the short molar teeth we have a short alveolar range and short rectangular ramus. This conformation is probably the primary cause of the mischief."

Again he says,—

"The condition under consideration may also arise from the tardy eruption of the molar teeth leaving the incisors to act for a time upon each other, as they do when from any cause the back teeth are lost. Then again, the incisors of the lower jaw may attain an unusual height, or they may project in an unusual degree, and produce the mischief. Or the result may be consequent upon a regular linear arrangement of large teeth in a jaw having a small alveolar base, in which case the teeth prior to their eruption will assume an unusual anterior obliquity."

But may we not very pertinently ask, What was the cause of the "excessive development," the "short molar teeth," the "short alveolar range," or the "short rectangular ramus?" The explanation can be regarded in no other light than a description of antecedent phenomena, but the origin of the deformity has not been reached.

In a paper read before the Odontological Society in London in 1864, Mr. Cartwright says,—

"Want of space in the bones of the jaws may be defined as the true cause of irregularity in the position of the teeth in the majority of instances;" and follows this statement immediately with an inquiry as "to a satisfactory explanation of this want of capacity in the jaws of people of certain communities."

He further says,—

"Irregularity is uncommon among many, if not most, aboriginal peoples and tribes, and also the inhabitants of particular districts and locations. Take the aborigines generally, North American Indians, Africans, Caffres included, and Asiatics.

"Irregularity is common in most highly civilized communities, and especially so among the upper and middle classes, and it is more constant among the inhabitants of towns than it is among the inhabitants of agricultural districts."

Mr. Cartwright then offers the hypothesis that this abnormality is due to a process of breeding, and brings forward Mr. Darwin's statements that the bones and plumage of birds become altered by such a process.

He further supports his position by the results of high breeding among animals, which is maintained by constant and careful selection of such as possess particular points and characteristics.

For instance, he says, "Take the horse and the ox, and consider the points which make up a thoroughbred animal, the small head and ears, the thin legs, small fetlocks and feet, the necks and bodies finely and symmetrically proportioned, and then the narrowness and comparative smallness of the maxillæ. From the results obtained by high breeding in animals, they might reasonably argue that small jaws might be a characteristic of breed in certain conditions of life. If they compared two types of human beings, represented by the upper class in one case and in the other case by a large class of which the prize-fighter furnished an apt example, they would find as a rule in the first: well-shaped lips, a small oral orifice, high and capacious fore-head, well-pronounced chin, ears small and neck long; the ankles, wrists, feet and hands small; with an expression in which the intellectual predominated over the animal;" while the other class, represented by the prize-fighter, presented exactly the opposite characteristics.

The hypothesis of Mr. Cartwright is not without reason; and yet it is not altogether an explanation of the present phenomena, for the laws which govern selective breeding in animals do not apply to man in any condition in which he is now found. These principles would, without doubt, produce precisely similar results if applied under similar conditions.

Regard man as an animal only; dismiss all cognizance of his intellect and his affections; mate him to the woman with sole reference to the physical development of the race, and it would take but a few generations to see disease and deformity swept from the face of the earth. But to call the intermarriage of families, whose brains have been stimulated to their highest capacity and whose physical and nervous systems have been deranged by the habits of modern civilization,—to call such a mixture high, or "selective breeding," is a perversion of the term. If the application of these principles produce a delicacy of form in the whole physique, we should reasonably expect to find a corresponding delicacy and refinement in the condition of the dental organs.

That the process of selective breeding should tend to, or end in, deformity, is manifestly inconsistent with its prime object, which is the elimination of everything which tends to degeneracy or deformity.

The characteristics pointed out by Mr. Cartwright in his illustrations from animals are conspicuously æsthetic.

We can conceive of no such force acting uniformly upon the whole physique, and producing such æsthetic results without its influencing equally the dental organs, and thus there would be not only no irregularity or abnormality, enhanced beauty,—more symmetrically formed, more symmetrically arranged, and more symmetrically related dental organs. We are therefore irresistibly led to the rejection of the theory of "high or selective breeding," in its true signification, as exerting any direct influence upon the malposition of the permanent teeth, and while the facts noticed by Mr. Cartwright are in consonance with other observers, we are forced to the conclusion that such abnormalities are not a necessary result of a higher civilization and refinement, and that they are only coincident and correlated thereto.

There is a kind of breeding which does undoubtedly produce abnormalities of the kind under discussion. It is a manifestation not uncommon in this country of mixed nationalities, but it can hardly be called *high* or *selective* breeding. The laws of inheritance, confirmed by common observation, show how constant is the mingling in the offspring of the traits of character and the peculiar features of two diverse races brought together in marriage.

This mixing, without blending or harmonizing, is productive of deformity in character and deformity in physique. Thus, so far as the jaws and teeth are concerned, they may exist in each parent in perfect symmetry,—in one parent the jaws and teeth are large, in the other parent both jaws and teeth are small; but each in its way is a normal development. If, now (and for which we can give no reason) the small jaw of one parent and the large teeth of the other appear in the offspring, deformity is sure to follow; and in any efforts made thereafter towards correction, these facts must be taken into consideration.

In examining the maxillæ of a child between the ages of four and seven years, with the external walls removed, so as to expose the developing permanent teeth, we shall be struck with the advanced stages of growth which the crowns have attained, and with the crowded and jumbled condition in which they are placed. Of such an exhibition Professor McQuillen made the following remark:

"When examining a series of jaws of different ages, arranged so as to show deciduous and permanent teeth, it is not a matter of surprise that there should be irregularity in the permanent set; but, when observing their crowded and irregular arrangement in the jaw prior to eruption, it is rather a matter of astonishment that they should ever assume a regular and symmetrical appearance."

In our investigation we will observe that while there is no transposition of these crowns—each one maintaining its individual locality—they nevertheless stand in almost every variety of position. They will be seen deflected within or without the line, twisted, lapping, and sometimes completely overriding one another.

In the specimen now before us the lower permanent central incisors have recently erupted, showing the age to be about seven years. Their predecessors are the only ones of the deciduous set which have been removed. The general contiguity of the temporary teeth shows that but little enlargement of the dental arch had taken place, while the presence of the crowns of all the permanent teeth (the third molars excepted) is an ample illustration of the crowded confusion and disorder which has been described. In the upper jaw the left central incisor is partially twisted; both of the lateral incisors are within the arch, and, if continued on the same line, would shut within the lower jaw.

The crowns of the canines override and are in front of the laterals, and in contact with the central incisors save by a thin partition of process.

The first bicuspids on each side are in close contiguity with the laterals. The crowns of all the bicuspids are either twisted or tipped in such a way as to show that some new direction must be given to nearly every tooth, or irregularity of the most pronounced character must ensue.

The general condition of the lower teeth is not dissimilar to the upper ones, and this example is not an exceptional case.

These crowns, we shall also observe, are of their full diameter, and that they are placed upon a maxillæ which at this age has not developed sufficiently to allow them to range side by side upon a true dental line; hence, they must remain in this crowned and irregular condition until the maxillæ itself has grown large enough to allow the change.

It is now an established fact that the development of the teeth and processes and the development of the jaws are two distinct and independent operations.

The maxillæ will go on in their growth until they have reached the measure prescribed by the Creator, whether a tooth develops or not, and likewise the teeth will grow into undiminished crowns and erupt either in order or disorder, whether the maxillæ increase or remain stunted.

I examined a few years since the mouth of the celebrated dwarf, "Tom Thumb." I found the teeth those of a man in individual size; but the maxillæ in harmony with the rest of his osseous system, were dwarfed. The result was what we should naturally expect,—a most marked malposition of the teeth,—so much so that he said he "had a double row of teeth all around." And this is uniformly the case with dwarfs where the whole physique is symmetrically dwarfed. The converse is also true of giants, except in those cases where the extraordinary stature is a characteristic of race.

To such an extent are these observations in accordance with both science and fact, that it is quite possible to judge of the nature of both these monstrosities, whether they be congenital, hereditary, or *lusus naturæ.* If, upon the examination of the fully-developed teeth of a giant, I found them large, regular, and in a well-formed arch, the whole in harmonious relation to his other enlarged features, I should have little hesitation in pronouncing him a congenital giant,—a large man by nature, and belonging to a family or race of giants. If, on the other hand, I found the teeth the size of those of a man of ordinary stature, and standing apart from each other in an enlarged jaw, I should pronounce him the result of forces which did not antedate his own existence, but continued their power long after the development of the teeth after their hereditary pattern had been completed. And in like manner a dwarf belonging to a race of small stature would not necessarily show irregularity or crowding of the teeth, but would be expected to show a reduction in size of the dental organs in consonance and in harmony with the type of his race.

This is but a confirmation of the theory of the independence of these organizations; the evidence from a variety of sources going to show that the forces which preside over the growth of the osseous system are separate and distinct from those which originate and develop the dental organs and that while in a normal state both teeth and jaws would grow in harmony; in an abnormal condition the teeth might be far in advance of the growth of the jaws or they might be equally retarded.

If the former, they must erupt in a crowded condition; if the latter, they would, other things being equal, be likely to be in line.

In recognizing an antagonism, I cannot but be impressed with a similar contest going on, more or less frequently, between the mental and the physical development,— between the brain and the body. In the normal state of man these two systems are found working in harmony; in the present refined or degenerate state, each is fighting for supremacy, with the odds in favor of the brain.

No one of extended observation will hesitate in believing that there is a faculty or power at work, modifying materially the physique of the present generation, altogether inexplicable by the two commonly asserted influencing power of climate, hygiene, or diet. One of the most alarming characteristics of the present age and the present civilization is found in the rapidity of its movements and the activity of its mind; in the larger aggregate of highly-organized and excessively developed nervous systems, and in the increasing tendency to nervous and brain diseases.

As the peculiarities of progenitors in mind, temperament and physique, are by nature stamped upon their offspring, we see a generation of children inheriting a tendency to a nervous exaltation which very slight favoring circumstances encourages and stimulates. This is unquestionably more noticeable in the centers of luxury in this country than in any other portion of the civilized globe. Fathers who are under a mental strain to the very verge of insanity transmit that exaltation to their offspring. Children are no longer children, except in their immature physical development; and thus, if not in all instances an absolute intellectual precocity, we have relatively a mental and nervous development far in advance of the physical. Hence, if the mental is only up to the average of its years, we will find it associated with anything but a robust physique; and the contrast remains the same. I shall show that one of the manifestations of this precocious, highly-organized, and exquisitely-developed nervous system is its influence upon the development of the teeth, while the physical system is following in tardy but vain efforts to keep pace with it.

My proposition then is this: laying aside all cases that may be due to an inherited tendency to follow or exaggerate some given type, together with those which are manifestly due to forces operating only after eruption; the primary cause, so far as the individual is concerned, of any general disturbance in the development of the permanent teeth, showing itself particularly in their malposition, is directly traceable to a lesion or innervation of the trigeminal nerve; that it is an interference, more or less prolonged, with one of the prominent functions of that nerve, and operating at its origin; that while I know of no way to prove this assumption by any examination, microscopical or otherwise while the nerve-center is under this influence, it is nevertheless sufficiently proven by secondary phenomena which could only have originated from such a source.

The function of the trigeminus thus stimulated or interrupted is that which supports, regulates, and governs the nutrition of the tissues to which its terminal branches are distributed.

That such a lesion or innervation would be likely to produce such result, is clearly foreshadowed in the following statement made by the late Professor Anstie in one of his lectures on the fifth nerve, as follows:

"The nervous center in which the trigeminus is implanted is, of all nervous centers, the one which, in the human subject, is most liable to congenital imperfection of the kind which necessitates a break-down in its governing functions at special crises in the development of the organism."

TO BE CONTINUED.

